# Exome sequencing identifies somatic point mutations associated with acquired endocrine resistance in breast cancer cell lines

**DOI:** 10.1186/1753-6561-6-S6-P35

**Published:** 2012-10-01

**Authors:** Natasja S Ehlers, Zhu Shi Da, Daniel Elias, Xue Lin, Jian Li, Christina Bjerre, Nils Brunner, Lars Bolund, Wang Jun, Ramneek Gupta, Henrik J Ditzel

**Affiliations:** 1 Sino-Danish Breast Cancer Research Centre; 2 Center for Biological Sequence Analysis, Department of Systems Biology, Technical University of Denmark, Building 208, DK-2800 Kongens Lyngby, Denmark; 3 BGI-Shenzhen, Beishan Industrial Zone, Yantian District, Shenzhen 518083, China; 4 Department of Cancer and Inflammation Research, Institute of Molecular Medicine, University of Southern Denmark, Winslowsvej 25, DK-5000 Odense, Denmark; 5 Department of Human Genetics, Aarhus University, Wilhelm Meyers Allé 4, DK-8000 Aarhus C, Denmark; 6 Section for Pathobiology, Department of Veterinary Disease Biology, Faculty of Life Sciences, University of Copenhagen, Dyrlægevej 88, I. DK-1870 Frederiksberg C, Denmark

## Background

Endocrine therapy is an effective treatment of estrogen receptor-positive (ER+) breast tumors, significantly reducing mortality. However, approximately 30% of patients receiving adjuvant endocrine therapy will experience recurrence within a 15-year period. The mechanisms of endocrine resistance are poorly understood. Understanding the underlying genetic diversity of breast cancers responding differently to endocrine therapy is important for the development of more optimal and individualized treatments strategies.

## Materials and methods

In the current study, a panel of isogenic MCF-7-derived human breast cancer cell lines [1-3] that are resistant to tamoxifen only, or to both tamoxifen and fulvestrant, respectively, were analyzed for mutations through exome sequencing and compared with the exome of the parental cell line. In addition, global gene expression levels for the same panel of cell lines were generated. Detected variations were integrated with gene expression profiles and analyzed in the context of prior knowledge of drug action and genes associated with resistance to endocrine therapies as identified by extensive literature curation.

## Results and conclusion

A small panel of somatic point mutations potentially associated with acquired endocrine resistance were identified. Future experimental validation will reveal which of the detected mutations that are causatively involved in resistance to endocrine therapy.
